# Supplementary material for: Anomalous entropy-driven kinetics of dislocation nucleation
Source: Nat Commun. 2025 Jan 21;16:912. doi: 10.1038/s41467-025-56272-4 (PMC11751456; doi:10.1038/s41467-025-56272-4)
Supplement: Supplementary file 1 — Supplementary Information [file 41467_2025_56272_MOESM1_ESM.pdf]

Supplementary Information

Anomalous entropy-driven kinetics of dislocation nucleation

Soumendu Bagchi* and Danny Perez

Theoretical Division, Los Alamos National Laboratory, NM, USA, 87545
*Current Affiliation: Center for Nanophase Materials Sciences, Oak Ridge National Laboratory, TN, USA, 37831

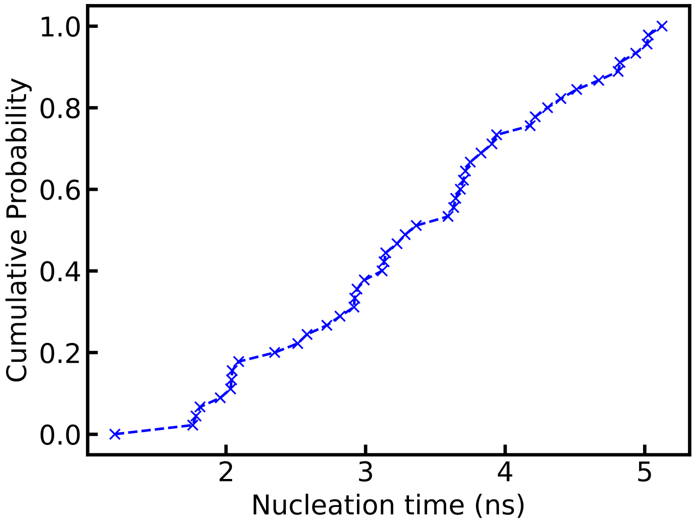

Supplementary Figure 1: Cumulative distribution of nucleation times of a leading partial dislocation loop from surface step of Cu EAM model under 2% compression subjected to temperature rate of 10K/ns starting from 600K for 50 randomly initiated MD runs.

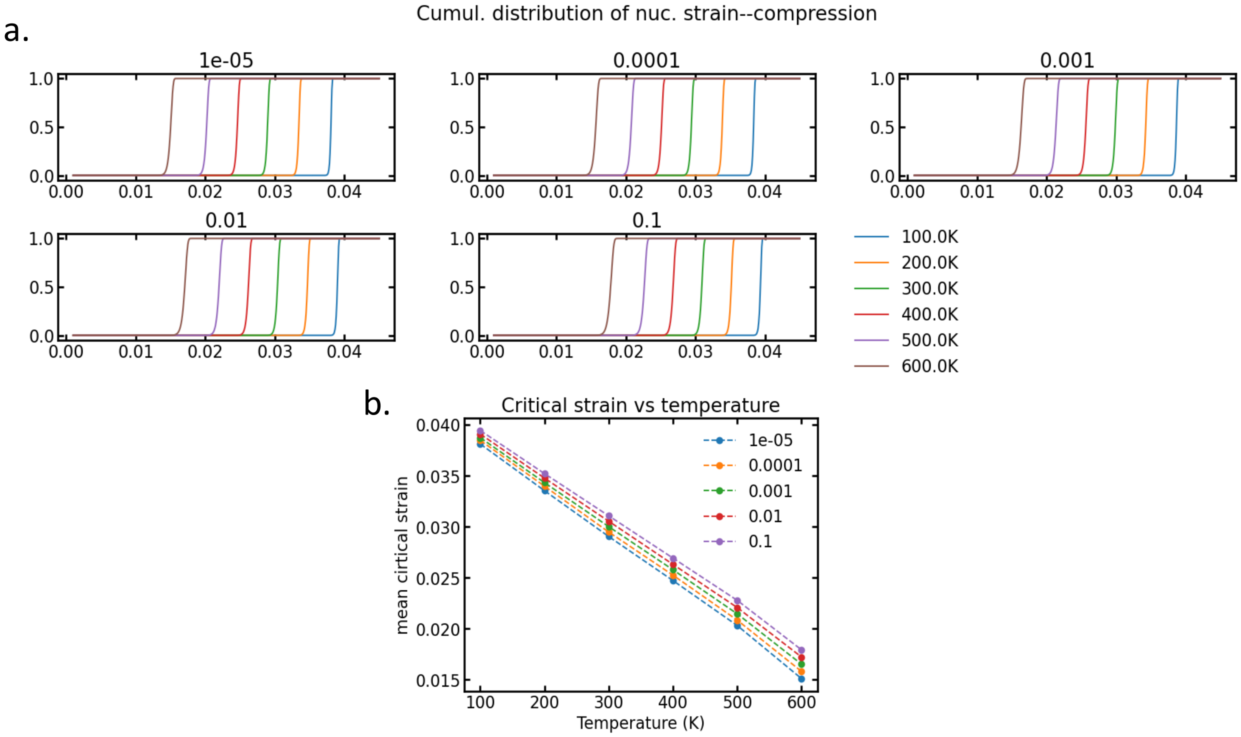

Supplementary Figure 2: Uniaxial compression nucleation strain analysis: **a.** Cumulative distribution of nucleation strain under various temperatures ranging from 100K to 600K and strain-rates ranging from 10^{-5} /s to 0.1 /s. **b.** Mean/critical nucleation strain decreases with temperature for different strain rates.

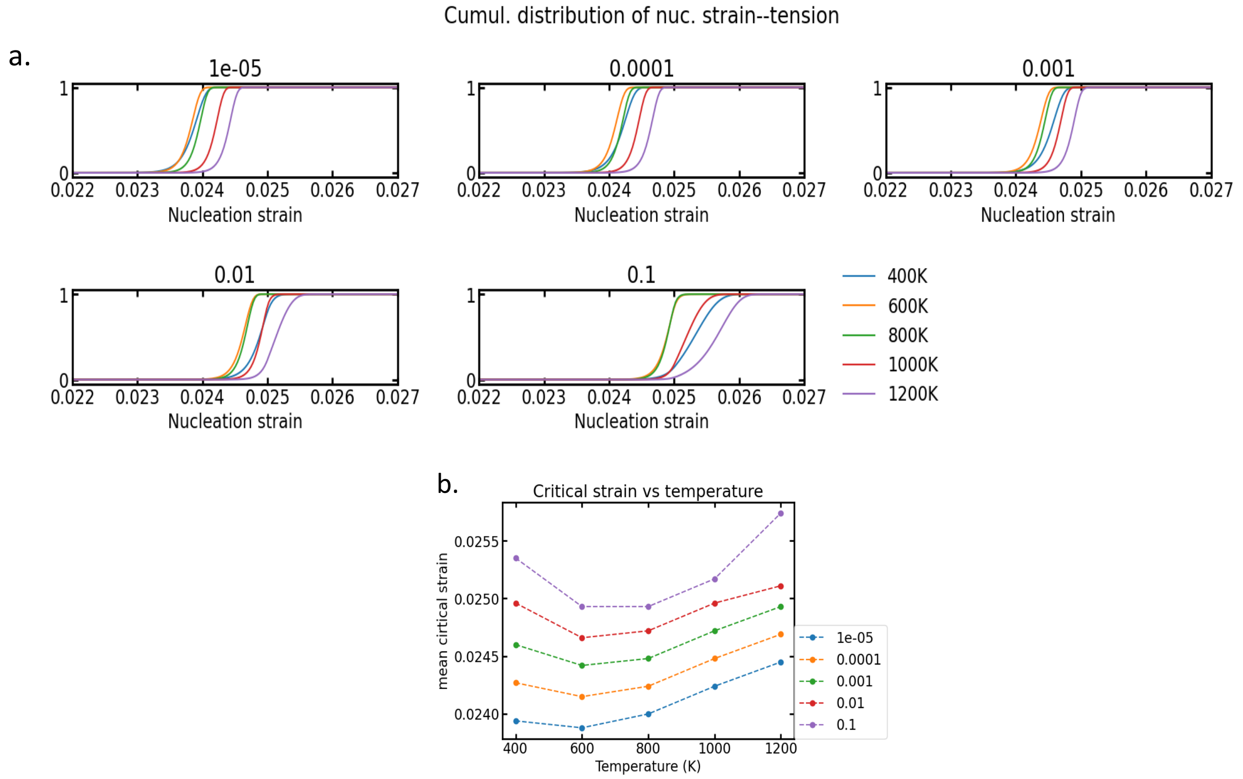

Supplementary Figure 3: Anti-Arrhenius nucleation at under uniaxial tensile strain: **a.** Cumulative distribution of nucleation strain under various temperatures ranging from 400K to 1200K and strain-rates ranging from 1^{-5} /s to 0.1 /s. **b.** Mean/critical nucleation strain (computed for distribution range 0.1 to 0.9) first decrease and then increases with temperature under compression.

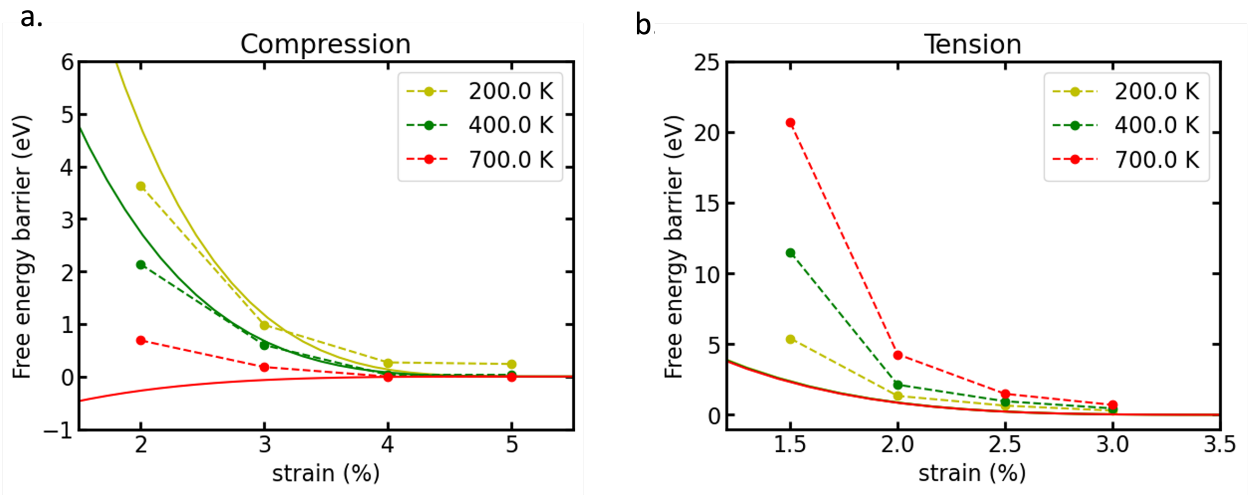

Supplementary Figure 4: Meyer-Neldel (MN) fits obtained for our compression (a.) and tension (b.) free energy barriers. With a fitted value of $T_m = 695K$, it is clear that MN predictions of the barrier deviates significantly with low strain and high temperatures (>$695K$). The MN model is not designed to capture anti-Arrhenius nature of barrier variation (i.e., entropic suppression), as shown in (b).

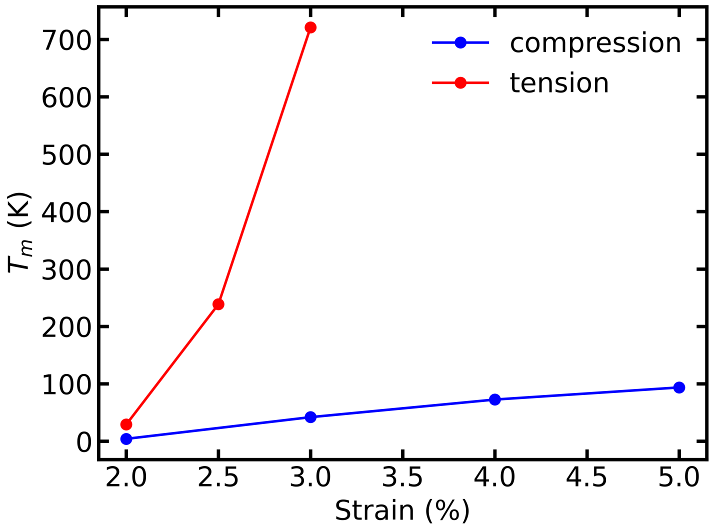

Supplementary Figure 5: Variation of fitting parameter T_m with strain as presented in our simplified analytical variational TST rate model. The rate predictions from this model are depicted in the main text c.f. Figs 2b and 3c.

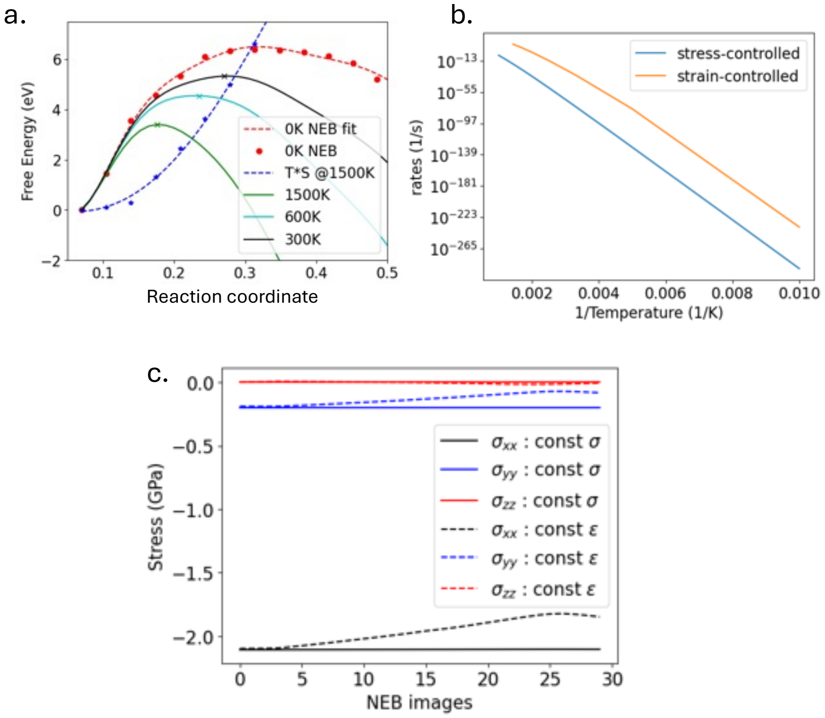

Supplementary Figure 6: Stress controlled analysis of (Gibbs) free energy and rates: For comparison with constant strain NEB and (Helmholtz) free energy analysis (c.f. Fig. 2 in main text), using a perturbative approach (c.f. Methods) we compute the Gibbs free energy, enthalpy (red) and entropy (blue) variation along the MEP in (a). While qualitatively identical, due to lack of local strain energy relaxation associated

with the stress-controlled nucleation pathway, the anharmonic entropic effect on rates is reduced than in case of strain-controlled nucleation (b). This is further clarified in (c) showing the change of stress through the replicas along the MEP for both stress (solid) and strain (dashed) controlled NEB results. Around 0.25GPa of relaxation in xx (loading) direction could lead to additional local entropy contribution in the strain-controlled nucleation process. This leads to a quantitative difference in the rates, while the variational free energy barrier along MEP for both cases, leads to overall same qualitative trend. Note, that the HTST prefactor at the enthalpy saddle is still very large (10^{39} /s) than standard prefactors.

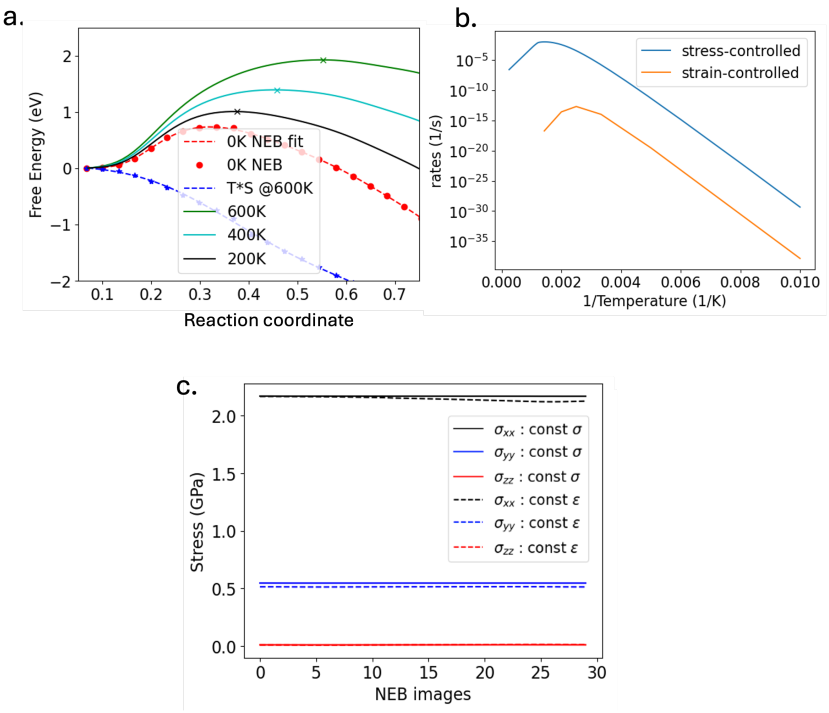

Supplementary Figure 7: Gibbs free energy and rate estimates similar to Supple. Fig. 6, but under tensile loading. As the differences between the stress components e.g., xx (loading direction--black), yy (blue) and zz (red) are relatively smaller than in the case under compression, the rates vary by 5 orders of magnitude. In line with the compression case, the results for both strain and stress-controlled loading, show considerable entropic effects, especially qualitatively similar anti-Arrhenius behavior at high temperature. Similar to Supple. Fig. 6, minimum free energy path (a), nucleation rate dependence on temperature (b) and changes in stress state during nucleation along the pathway (c) are plotted.

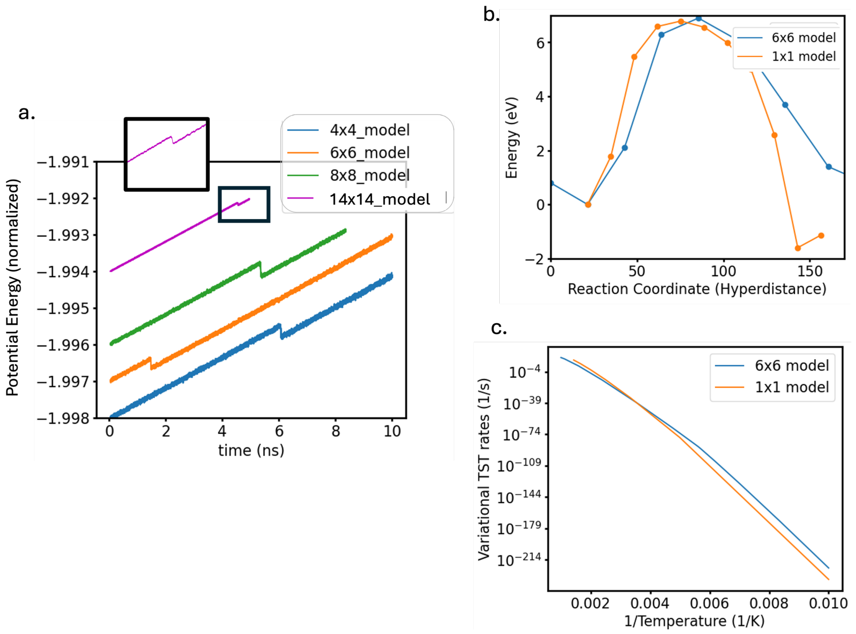

Supplementary Figure 8: Nucleation kinetics remain largely unaffected by model size variation (i.e. along in-plane x and y directions, where x is the loading direction and surface normal is along z-axis). Under 2% **compression**, as shown in 8a, different model sizes (4x4, 6x6, 8x8) upto 38 million atoms (14x14, with reference to our smallest model 1x1~ 0.18 million) stochastically nucleate when heated from 600K-700K over a duration of 10 ns. Furthermore, CI-NEB results in 8b, show that the potential energy barriers along the reaction path are also in the similar range (~6.8 eV). The variational rates due to anharmonic entropic contribution in the free energy leads to relatively unaffected nucleation rates (8c) and large prefactors for both the models.

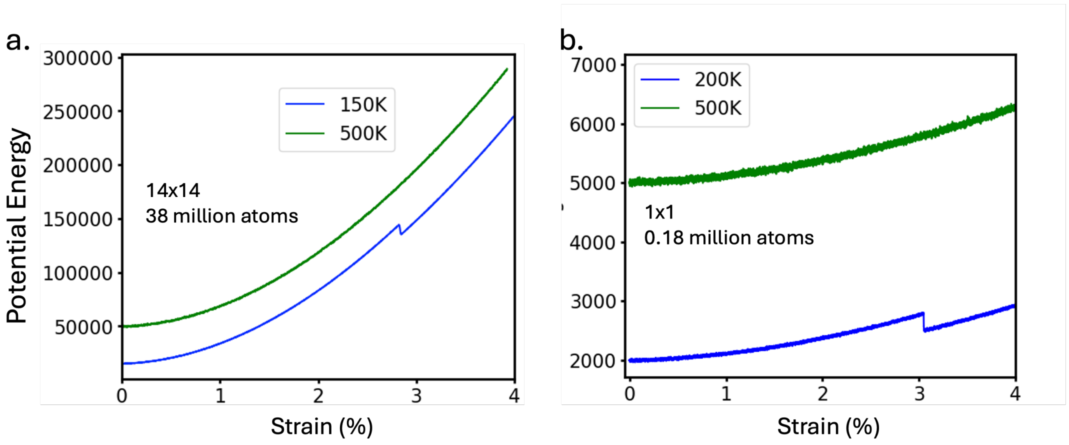

Supplementary Figure 9: Similar to Suppl. Fig. 8, under 2% **tension,** the anti-Arrhenius nucleation behavior reported in Section D. of the main text, remains consistent across model sizes ranging from 0.18 million (9a) to 38 million (9b). Low temperature nucleation at ~3% strain is suppressed at higher temperatures (e.g. 500K in 9a and 9b) in both the cases shown. This results from a negative entropy change which leads to an increase in or even emergence of free energy barriers. (c.f. Section D and Discussion in main text for details)
